# Health partnership research and the assessment of effectiveness

**DOI:** 10.1186/s12992-016-0181-9

**Published:** 2016-07-29

**Authors:** Dan Ritman

**Affiliations:** Tropical Health & Education Trust (THET), London, UK

**Keywords:** Health partnerships, Partnership effectiveness, Frameworks

## Introduction

Health partnerships are flourishing between institutions in the UK and low- and middle-income countries. Over the past five years, the Health Partnership Scheme (HPS), a UK government-funded programme managed by UK NGO THET, has supported health partnerships to undertake more than 200 projects in low- and middle-income countries [1]. All HPS-funded health partnerships, and most others, undertake monitoring and evaluation to generate high quality information and insights for effective management, stakeholder engagement, accountability and advocacy. There are many descriptive and reflective accounts of health partnerships in the literature (eg [2–5]) and a huge volume of grey literature in the form of project reports and evaluations.

With greater interest and investment comes higher profile and closer scrutiny. While this can manifest as pressure to generate evidence of short-term, measurable achievements, rather than long-term, sustainable impact [6], some health partnerships have responded by rigorously strengthening their evaluation and research activities. Emerging questions about the mechanisms, efficiency and effectiveness of health partnerships have prompted a stream of published evaluations and research papers from clinicians [1], social scientists [7], health systems researchers, economists and others. These questions relate to two topics: what health partnerships are, and what health partnerships do.

## Exploring what health partnerships do

Health partnerships undertake health workforce strengthening or related projects, and we can ask what the projects achieve in terms of health workforce strengthening [8], health systems strengthening [9] and patient outcomes, and how effective and cost-effective are the interventions they use, such as approaches to mentoring and training of trainers [10]. See [11] for a list of health partnership publications.

There are no generic answers to questions about the effectiveness of health partnership projects, given their extraordinary diversity of national and local contexts, institutions and people involved, issues tackled and interventions tried, although a sufficient body of research into individual partnership projects may help us identify areas in which health partnerships are most effective [12].

## Exploring what health partnerships are

Turning to what health partnerships are, we can research the common elements of the health partnership model, for instance:

- What makes an effective partnership, one able to design and manage high quality health workforce strengthening projects?
- How can we characterise the social capital generated by institutional and individual relationships?
- How do health worker volunteers compare to paid project staff, in terms of costs and benefits for both LMIC institutions and the UK health service?

These sorts of questions are crucial to understanding the value of the health partnership model and for making informed decisions about partnership funding. Evidence about the health partnership model will underpin (rather than answer) questions about the specific health service and health outcome improvements that individual health partnerships try to achieve.

## Partnership effectiveness

Several recent papers in Globalization & Health have evaluated the effectiveness of partnerships [13–16], both service delivery-focussed health partnerships, and health research partnerships and consortia. They share many characteristics: a concern with improving health outcomes; collaboration between individuals and institutions in high-income countries and low- and middle-income countries; an element of capacity-building; and the challenges of building strong relationships. We also note that the boundaries between health partnerships and research partnerships are not clear, since health partnerships often include a research element, and research partnerships often go on to deliver services [13, 15].

Research partnerships are arguably more equitable than service delivery partnerships, since researchers and research institutions are engaged in similar activities and have common expectations and goals, and offer similar contributions. Nonetheless it is striking how many of the topics highlighted in the research partnership papers [13–15] are applicable in health partnerships too, including the value of multidisciplinary teams, the challenges of developing a shared understanding across national and cultural boundaries, and the importance of individual relationships built over time.

Hill et al. [13] present the history of the Kenya National Retinoblastoma Strategy, a multidisciplinary group of health workers, researchers, health service users, and government and NGO staff, formed to explore and tackle the constraints to high quality treatment of retinoblastoma. Formed in 2008, the group has moved through phases of planning, capacity building and research and innovation. Hill et al. evaluate the group against the Swiss Commission for Research Partnerships with Developing Countries (KPFE)’s Framework for transboundary research partnerships [17]. They highlight the value of the multidisciplinary group for taking a holistic approach to the issue, noting the corresponding challenge of reconciling different points of view. Annual meetings have been crucial opportunities for the group to build relationships and find consensus, to undertake significant pieces of work and to reflect. The study also highlights the group’s flexibility in adapting activities to circumstances.

Larkan et al. [14] review the Centre for Global Health, Trinity College Dublin’s partnerships with a range of institutions in more than 40 countries. Inviting reflections from members of the partnerships, they highlight the complex historical and contextual factors in understanding and managing partnerships, and use thematic analysis to identify and describe in detail seven guiding principles of effective partnerships.

Elmusharaf et al. [15] present and reflect on the achievements of the Connecting health Research in Africa and Ireland Consortium (ChRAIC) in Sudan, one member of the consortium. ChRAIC helped to establish a national research team in Sudan which began researching access to maternal health services, with international support for building capacity and institutional linkages. Meanwhile, a Sudanese student joined a ChRAIC-established PhD programme in Ireland on health system research. The PhD student engaged deeply with the work of the research team in Sudan, using participatory research techniques and developing effective maternal health innovations including innovative participatory health education. Elmusharaf et al. do not use or generate a framework for partnership effectiveness but they do highlight the importance to the project of this engagement, which they attribute to the commitment of the PhD student and the lead partner in Sudan, and to the parallel development of the PhD research and the research team; they also highlight the strong need for support perceived in Sudan, which influenced attitudes to ChRAIC.

Ramaswamy et al. [16] describe the partnership model used by Kybele, a US-based nonprofit organisation that works to improve childbirth safety in middle-income countries. Kybele works in partnership with tertiary hospitals in those countries, to strengthen clinical capacity and systems in their obstetric, anaesthetic and paediatric departments, and then throughout the country. Kybele has identified a generic sequence of activities for partnership working and adapted it to its own principles to generate a model used for service delivery partnerships.

## Partnership effectiveness frameworks

The frameworks developed or used by these researchers can be used to assess other partnerships, and as guidelines for partnership strengthening. The variety of terminology and different levels of abstraction make it hard to compare them, but unsurprisingly there are common themes (see Table 1); as well as aspects stressed by some but not all frameworks.

**Table 1:** Examples of common approaches to partnership working in four frameworks

Swiss Commission for Research Partnerships with DCs’ framework for transboundary research partnerships ([17], cited in [13])	Framework for Successful Research Partnership in Global Health [14]	Kybele Partnership Model (cited in [16])	THET’s Principles of Partnership [18]
Set the agenda together; Interact with stakeholders	Focus (including common goals and programme, vision)	Develop local solutions based on assessment of need and capacity	Strategic (including plans linked to identified needs); Respectful and reciprocal (including mutual engagement with each other’s needs and ideas)
Clarify responsibility	Leadership (including delegation of roles, management)	Ensure that a champion is selected who is committed to a partnership	Organised and accountable (including governance structures)
Promote mutual learning	[not explicit]	M&E integrated into all Kybele programs	Committed to joint learning

A recent study (Edwards S, forthcoming) compared the experience of one health partnership to the THET Principles of Partnership [18]; largely validating it but highlighting some areas of difference. Framework development, validation and application are essential for strengthening our understanding of the value of partnerships in global health, and point to the importance of research in this area.

The plethora of frameworks – and there are others in the literature – highlights the potential for duplication of effort and the importance of collaboration in the emerging field of health partnership research. THET recently convened a meeting of researchers, practitioners and policy-makers to consider questions, methods, challenges and models in health partnership research, and their implications for research capacity and funding. We will publish a health partnership research agenda in due course that outlines these considerations for health partnership practitioners and policy-makers.

## Abbreviations

ChRAIC, Connecting health Research in Africa and Ireland Consortium; HPS, Health Partnership Scheme; KPFE, Swiss Commission for Research Partnerships with Developing Countries; LMIC, low and middle income countries; NGO, non-governmental organisation; THET, Tropical Health & Education Trust
